# Self-Reported Side Effects and Adherence to Antiretroviral Therapy in HIV-Infected Pregnant Women under Option B+: A Prospective Study

**DOI:** 10.1371/journal.pone.0163079

**Published:** 2016-10-19

**Authors:** Tamsin Phillips, Annibale Cois, Robert H. Remien, Claude A. Mellins, James A. McIntyre, Greg Petro, Elaine J. Abrams, Landon Myer

**Affiliations:** 1 Division of Epidemiology & Biostatistics, University of Cape Town, Cape Town, South Africa; 2 Centre for Infectious Diseases Epidemiology & Research, University of Cape Town, Cape Town, South Africa; 3 HIV Center for Clinical and Behavioral Studies, New York State Psychiatric Institute, Columbia University, New York, NY, United States of America; 4 Anova Health Institute, Johannesburg, South Africa; 5 Department of Obstetrics & Gynaecology, University of Cape Town, Cape Town, South Africa; 6 New Somerset Hospital, Cape Town, South Africa; 7 ICAP, Columbia University, Mailman School of Public Health, New York, NY, United States of America; 8 College of Physicians & Surgeons, Columbia University, New York, NY, United States of America; University of Maryland School of Medicine, UNITED STATES

## Abstract

**Background:**

Antiretroviral therapy (ART) regimens containing efavirenz (EFV) are recommended as part of universal ART for pregnant and breastfeeding women. EFV may have appreciable side effects (SE), and ART adherence in pregnancy is a major concern, but little is known about ART SE and associations with adherence in pregnancy.

**Methods:**

We investigated the distribution of patient-reported SE (based on Division of AIDS categories) and the association of SE with missed ART doses in a cohort of 517 women starting EFV+3TC/FTC+TDF during pregnancy. In analysis, SE were considered in terms of their overall frequency, by systems category, and by latent classes.

**Results:**

Overall 97% of women reported experiencing at least one SE after ART initiation, with 48% experiencing more than five SE. Gastrointestinal, central nervous system, systemic and skin SE were reported by 81%, 85%, 79% and 31% of women, respectively, with considerable overlap across groups. At least one missed dose was reported by 32% of women. In multivariable models, ART non-adherence was associated with systemic SE compared to other systems categories, and measures of the overall burden of SE experienced were most strongly associated with missed ART doses.

**Conclusion:**

These data demonstrate very high levels of SE in pregnant women initiating EFV-based ART and a strong association between SE burden and ART adherence. ART regimens with reduced SE profiles may enhance adherence, and as countries expand universal ART for all adult patients, counseling must include preparation for ART SE.

## Background

With the recent adoption of Option B+ for prevention to mother-to-child HIV transmission (PMTCT) there have been rapid increases globally in the use of antiretroviral therapy (ART) by HIV-infected pregnant and breastfeeding women.[1] Following initiation of ART, successful treatment implementation and continued adherence to lifelong therapy is a widespread concern, emerging as a particular issue for women initiating ART during the perinatal period.[2,3] In turn, understanding adherence under Option B+ and the determinants of non-adherence, is a major priority both for individual and program level PMTCT outcomes.

Medication side effects (SE) are commonly thought to influence treatment adherence.[4–6] Efavirenz (EFV)-containing ART regimens are currently recommended by the WHO as first line treatment. They are widely used in resource-limited settings and are generally considered to be better tolerated than previous NNRTI regimens.[7–10] However, specific SE associated with EFV are well known, particularly neuropsychiatric SE such as dizziness and vivid dreams.[11,12] In general, SE from current first line therapy are thought to be relatively short lived and improve as patients continue on ART. However, these early SE may disrupt initial implementation of the regimen, and there is some evidence that early treatment adherence is important to set the course of long term adherence and treatment success.[13] Early adherence, particularly during pregnancy, is required to ensure rapid viral suppression, reduced transmission risk and improved maternal health outcomes.

Experience of SE has been associated with non-adherence and discontinuation of ART in general adult populations using various regimens.[12,14–19] However, few studies focus specifically on EFV-containing regimens and treatment adherence. One study in a small general adult cohort in South Africa, the majority on EFV-based regimens, found that experience of symptoms was associated with lower self-reported adherence scores.[17] Fear and experiences of SE have been mooted as threats to ART adherence and continued ART use both in general adult patients as well as in pregnant and postpartum women.[3,15,20–25]

The role of SE in ART adherence warrants special attention in pregnant and postpartum women. To date evaluations of SE in pregnant populations have been mostly limited to clinician-reported adverse events, primarily in trial settings.[26–29] Yet patients’ experiences of SE may differ from clinician-reported adverse events.[7,18,30] While data on SE under Option B+ are few, country-level data show high rates of early loss to follow-up under Option B+, particularly among women with higher baseline CD4 cell counts, which may be attributable in part to ART SE.[31,32] The experience and tolerance of ART SE in relatively healthy individuals, commonly the case in pregnant women initiating ART under Option B+, may differ from that of individuals with more advanced HIV disease.[30,33–35] Also, in the context of pregnancy, ART initiation is frequently fast-tracked to maximize the chances of reaching viral suppression by delivery. There may be limited time to counsel patients on common SE and their management, and lack of preparation for SE has been suggested as a risk factor for poor adherence in the context of Option B+.[23]

Despite the importance of these issues, little is known about experience of SE and the relationship with adherence after ART initiation in pregnancy, particularly with first line EFV-containing regimens in the context of Option B+. We examined the occurrence and patterns of SE during pregnancy among women initiating EFV-based ART, and investigated the relationship between reported SE and reported missed ART doses.

## Methods

### Population

This analysis draws on data from a large multi-phase trial evaluating strategies for delivering HIV care and treatment services during pregnancy and the postpartum period (The MCH-ART study, https://clinicaltrials.gov/ct2/show/NCT01933477).[36] The study took place in a large public sector antenatal clinic (ANC) in Gugulethu, Cape Town, South Africa. This setting is characterized by high levels of poverty and HIV with a local antenatal HIV seroprevalence of 33%.[37] Local public sector health services have provided free ART services since 2004 and from 2012 ART was delivered within the ANC. From July 2013, all HIV-infected pregnant women were eligible to start lifelong ART regardless of CD4 cell count or clinical stage (Option B+).[38]

Consecutive HIV-infected, ART-eligible pregnant women 18 years and older making their first ANC visit during the current pregnancy were enrolled into the MCH-ART study between April 2013 and June 2014. Overall, 526 women were enrolled under Option B+ which started on 1 July 2013. Women started a first line regimen of EFV (600mg), emtricitabine (FTC)/ lamivudine (3TC) (300mg) and tenofovir (TDF) (300mg) (EFV+FTC/3TC+TDF), provided as a fixed dose combination. ART initiation and follow-up was conducted by nurse-midwives within the ANC. ART was usually initiated on the day of the first antenatal visit or within two weeks, with routine follow-up visits occurring monthly for the first four months on treatment and one to two monthly thereafter. ART counselling was provided by trained counsellors before initiation and at each follow-up visit.

The cohort was followed through study assessments conducted separately from routine care. Participants completed an interviewer-administered questionnaire at the time of enrolment, up to twice more during pregnancy, and once immediately after delivery. Each study assessment included structured questionnaires on ART use, and at the first postpartum study visit self-reported SE experienced since ART initiation were assessed. This study was conducted in accordance with the ethical standards of the 1964 Helsinki declaration and its later amendments, and was approved and conducted in accordance with the standards of the Human Research Ethics Committee of the University of Cape Town and the Columbia University Medical Centre Institutional Review Board. All participants provided written informed consent.

### Measures

A structured questionnaire based on Division of AIDS (DAIDS) Tables for grading adverse events[39] was used to collect information on SE experienced, including gastrointestinal (GIT) (nausea/vomiting, appetite change, diarrhea, other GIT), central nervous system (CNS) (headache, dizziness, unusual dreaming, other CNS), skin (rash, other skin), systemic (fatigue, fever/sweats, non-specific pain, other systemic) SE. The collected information was codified using a set of 14 binary variables for the reporting of each SE from ART initiation through to the first postpartum study visit.

Missed doses in the preceding 30 days were reported at each visit. The average number of missed ART doses was calculated by summing the reported missed doses across all study visits between ART start and the first postpartum visit. The sum of missed doses was divided by the sum of the reference period (the maximum number of visits on ART completed was three, resulting in a maximum reference period of 90 days) and multiplied by 30 to obtain the average number of missed doses in a 30-day period. This was analyzed as both a continuous and binary variable (any vs. no missed doses). A secondary outcome of a reported period of no treatment for 30 days or more at the time of the last assessment was also evaluated.

### Analysis

This secondary analysis of data from the MCH-ART study excluded women enrolled under the Option A policy as well as women who did not have data available on side effects in pregnancy (n = 9). Women were included regardless of mode of delivery. Data were analyzed using Stata Version 12.0 (Stata Corporation, College Station, Texas, USA) and MPlus Version 7.3 (Muthén & Muthén, Los Angeles, CA). Variables were described using medians (with interquartile ranges, IQR) and proportions with (95% confidence intervals, CI). We used rank-sum and chi-square (replaced in the case of sparse data by Fisher’s exact) tests to examine differences in central tendencies and proportions, respectively. Three different constructs were used to examine the patterns of SE reported by participants and the associations with missed ART doses:

- *Systems categories*: A classification based on Division of AIDS (DAIDS)[39] categories was used to divide reported SE into groups of gastrointestinal (GIT), central nervous system (CNS), skin and systemic SE. Assignment to these categories was not mutually exclusive.
- *Cumulative number of SE*: Subjects were grouped according to the number of different types of SE experienced since ART initiation (from no SE reported to up to 14 different SE).
- *Latent SE class*: Latent Class Analysis (LCA) was used to classify individuals into mutually exclusive groups (latent classes) characterized by different pattern of SE, allowing for patterns that may not be solely systems-based (as assumed in [a] above). LCA, a statistical method for inferring unmeasured class membership using a set of measured variables as imperfect class indicators, has been used to identify underlying patterns of signs and symptoms in other contexts.[40–43] Here the binary variables representing presence/absence of different SE since ART initiation were used as indicators, and the optimal number of latent classes was determined by comparing the fit of models with different number of classes according to statistical information criteria together with the results of the bootstrap likelihood ratio test.[44] Individuals were assigned to each class according to the maximum posterior probability. The LCA process is described in S1 and S2 Tables.

Multiple logistic regression was used to investigate adjusted associations between reported SE (according to the three different classifications described above) and reported missed ART doses. Covariates with associations at p≤0.10 in the bivariate models were included in the multivariable models. When analyzing predictors of latent class membership, the probability of misclassification of individuals was taken into account in the estimation, using the three-step approach described by Asparouhov and Muthén.[45]

## Results

Overall, 517 women who initiated ART in pregnancy under Option B+ were included in this analysis (Table 1). The median age was 28 years, 74% of women had not completed secondary school and 63% were unemployed. At ART initiation, the median CD4 cell count was 361 cells/μL (IQR 244–539 cells/μL) and the median gestational age was 21 weeks (IQR 16–27 weeks). HIV diagnosis occurred during the current pregnancy for 55% of women and 28% had used some form of antiretroviral previously (predominantly short-course PMTCT regimens). All women received folic acid, calcium carbonate and iron supplements during antenatal care, and 6 women were treated for tuberculosis during pregnancy. At the time of the SE assessment postpartum (median, 7 days after delivery) the median duration of ART use was 19 weeks (IQR 12–23 weeks).

**Table 1:** Demographic characteristics of 517 women initiating ART during pregnancy included in the study, by reported side effects (SE). All cells are N (%) unless otherwise specified.

	All women	Any SE	>5 SE	1–5 SE	No SE	p-value (any vs none)
**Number of women**	517	502(97)	250(48)	252 (49)	15(3)	-
**Median age (IQR)**	28(24–32)	28(25–32)	28(24–32)	28(25–32)	33(23–34)	0.364
**Socioeconomic status**						
**lowest**	197(38)	190(38)	103(41)	87(35)	7(47)	0.758
**medium**	152(29)	149(30)	68(27)	81(32)	3(20)	
**highest**	168(33)	163(32)	79(32)	84(33)	5(33)	
**Education level**						
**Finished high school**	134(26)	130(26)	59(24)	71(28)	4(27)	1.000
**Did not finish high school**	383(74)	372(74)	191(76)	181(72)	11(73)	
**Employment status**						
**Employed**	193(37)	187(37)	91(36)	96(38)	6(40)	0.794
**Not employed**	324(63)	315(63)	159(64)	156(62)	9(60)	
**Relationship status**						
**Married/cohabiting**	198(38)	194(39)	88(35)	106(42)	4(27)	0.427
**Not married/cohabiting**	319(62)	308(61)	164(65)	144(58)	11(73)	
**Median gravidity (IQR)**	2(2–3)	2(2–3)	2(2–3)	2(2–3)	3(2–3)	0.183
**Primigravida**	93(18)	91(18)	50(20)	41(16)	2(13)	1.000
**Mulitgravida**	424(82)	411(82)	200(80)	211(84)	13(87)	
**Timing of HIV diagnosis**						
**In the current pregnancy**	286(55)	278(55)	135(54)	143(57)	8(53)	1.000
**Prior to this pregnancy**	231(45)	224(45)	115(46)	109(43)	7(47)	
**ARV history**						
**ARV naïve**	374(72)	365(73)	186(74)	179(71)	9(60)	0.386
**Previous PMTCT**	126(24)	120(24)	53(21)	67(27)	6(40)	
**Previous ART**	17(3)	17(3)	11(4)	6(2)	0(0)	
**Median CD4 cell count at ART initiation (IQR)** ^**§**^	361(244–539)	359(242–536)	337(225–507)	390(271–553)	502(294–635)	0.104
**CD4<200**	85(17)	85(18)	52(21)	33(14)	0(0)	0.200
**CD4 200–349**	150(30)	145(30)	76(31)	69(29)	5(33)	
**CD4≥350**	265(53)	255(53)	115(47)	140(58)	10(67)	
**Median gestation(weeks) at ART initiation (IQR)**	21(16–27)	21(16–27)	20(15–25)	22(17–29)	23(13–21)	0.360
**Median weeks on ART (IQR)**	19(12–23)	19(12–23)	19(15–24)	17(10–23)	9(4–21)	0.012

^§^17missing CD4 count

### Prevalence of side effects

**Fig 1:**
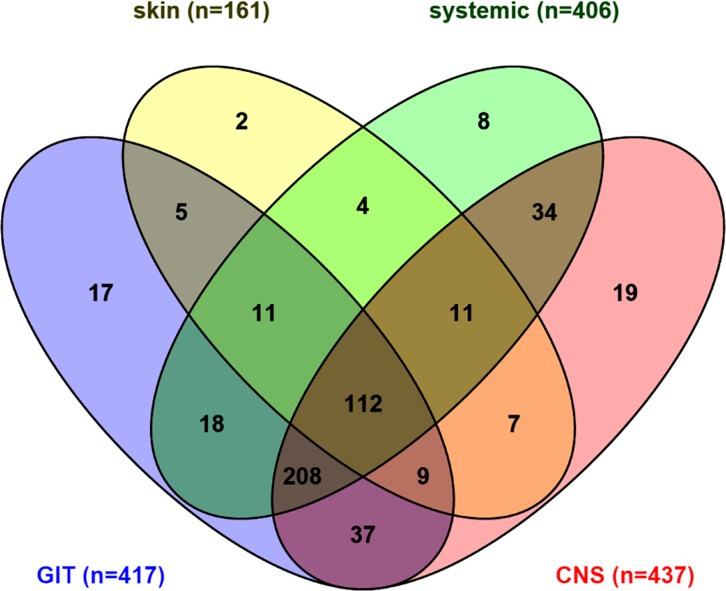
Venn diagram of number of women reporting each side effect by system category.

Overall, 97% of women (n = 502) reported experiencing at least one SE on ART during pregnancy. Only median duration on ART differed between women who did and did not report SE (19 and 9 weeks, respectively; p = 0.012) (Table 1). The median number of different SE experienced was five (IQR 3–7; max 12) and 91% of women reported two or more different SE. GIT, CNS, systemic and skin SE were reported by 81%, 85%, 79% and 31% of women, respectively. There was considerable overlap in SE reporting (Fig 1) with 22% of women (n = 112) experiencing at least one SE from all four categories, and 40% (n = 208) reporting GIT, CNS and systemic side effects. The distributions of each SE construct by socio-demographic subgroups are displayed in S3 Table.

### Latent side effect classes

**Fig 2:**
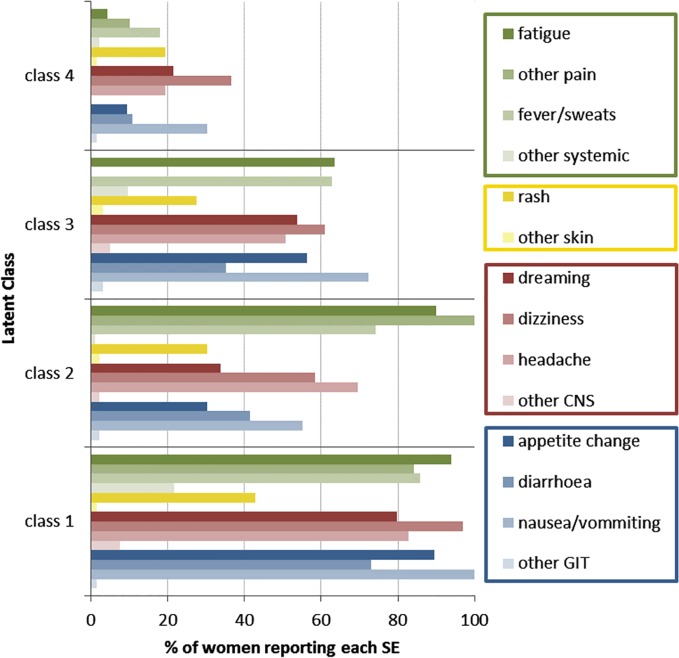
Proportion of women reporting each side effect by latent side effect (SE) classes; systems-based SE categories are shown in green (systemic SE), yellow (skin SE), red (central nervous system SE) and blue (gastrointestinal SE).

Four distinct patterns of SE identified using LCA are shown in Fig 2 and described in Table 2. One group of women, comprising 26% of the cohort, reported experiencing most of the assessed SE (here referred to as Class 1). A second group (Class 2, 17% of the cohort) frequently reported experiencing systemic SE but reported experiencing CNS, GIT and skin SE less commonly. Class 3 (30% of women) reported experiencing all types of SE less commonly (including systemic SE), while a fourth class (27% of women) reported experiencing few SE.

**Table 2:** Reported side effects among 517 women following ART initiation during pregnancy in Cape Town, South Africa. All cells are N (%) unless otherwise specified.

	All women	Missed doses in 30 days (on average)				Discontinued ART		p-value (any versus no missed dose)
		None	1–3	4 or more	Any missed	on ART	Stopped ART	
**Number of women**	**517**	**351**	**145**	**21**	**166**	**511**	**6**	
**Any side effect reported**	**502(97)**	**336(96)**	**145(100)**	**21(100)**	**166(100)**	**496(97)**	**6(100)**	**0.003**
**Median number of reported SE (IQR)**	***5(3–7)***	***5(2–7)***	***6(4–8)***	***8(5–9)***	***6(4–8)***	***5(3–7)***	***8(7–9)***	***<0*.*001***
*No reported SE*	15(3)	15(4)	0(0)	*0(0)*	*0(0)*	15(3)	0	*<0*.*001*
*Only 1 reported SE*	32(6)	28(8)	3(2)	*1(5)*	*4(2)*	31(6)	1(17)	
*More than 1 reported SE*	**470(91)**	308(88)	142(98)	*20(95)*	*162(98)*	**465(91)**	**5(83)**	
**Any GIT SE**	**417(81)**	**269(77)**	**131(90)**	**17(81)**	**148(89)**	**412(81)**	**5(83)**	**0.001**
*Nausea or vomiting*	*337(65)*	*215(61)*	*107(74)*	*15(71)*	*122(73)*	*332(65)*	*5(83)*	*0*.*006*
*Diarrhea*	*204(39)*	*122(35)*	*73(50)*	*9(43)*	*82(49)*	*202(40)*	*2(33)*	*0*.*001*
*Appetite change*	*247(48)*	*147(42)*	*89(61)*	*11(52)*	*100(60)*	*244(48)*	*3(50)*	*<0*.*001*
*Other GIT*	*11(2)*	*6(2)*	*4(3)*	*1(5)*	*5(3)*	*11(2)*	*0*	*0*.*272*
**Any CNS SE**	**437(85)**	**289(82)**	**128(88)**	**20(95)**	**148(89)**	**431(84)**	**6(100)**	**0.045**
*Dizziness*	*327(63)*	*208(59)*	*101(70)*	*18(86)*	*119(72)*	*321(63)*	*6(100)*	*0*.*006*
*Unusual dreaming*	*250(48)*	*160(46)*	*75(52)*	*15(71)*	*90(54)*	*246(48)*	*4(67)*	*0*.*067*
*Headache*	*278(54)*	*182(52)*	*82(57)*	*14(67)*	*96(58)*	*273(53)*	*5(83)*	*0*.*203*
*All other CNS SE*	*20(4)*	*10(3)*	*9(6)*	*1(5)*	*10(6)*	*19(4)*	*1(17)*	*0*.*080*
**Any skin SE**	**161(31)**	**102(29)**	**48(33)**	**11(52)**	**59(36)**	**158(31)**	**3(50)**	**0.137**
*Rash*	*154(30)*	*95(27)*	*48(33)*	*11(52)*	*59(36)*	*151(30)*	*3(50)*	*0*.*049*
*All other skin SE*	*11(2)*	*8(2)*	*2(1)*	*1(5)*	*3(2)*	*11(2)*	*0*	*0*.*728*
**Any systemic SE**	**406(79)**	**256(73)**	**131(90)**	**19(91)**	**150(90)**	**401(78)**	**5(83)**	**<0.001**
*Fever or sweats*	*303(59)*	*191(54)*	*96(66)*	*16(76)*	*112(67)*	*299(59)*	*4(67)*	*0*.*005*
*Other nonspecific pain*	*215(42)*	*126(36)*	*75(52)*	*14(67)*	*89(54)*	*210(41)*	*5(83)*	*<0*.*001*
*Fatigue*	*310(60)*	*190(54)*	*104(72)*	*16(76)*	*120(72)*	*305(60)*	*5(83)*	*<0*.*001*
*All other systemic SE*	*48(9)*	*30(9)*	*14(10)*	*4(19)*	*18(11)*	*48(9)*	*0*	*0*.*401*
**Class 1 (high SE)**	133(26)	74(21)	31(28)	28(51)	59(36)	130(25)	3(50)	<0.001
**Class 2 (moderate SE, high systemic)**	89(17)	55(16)	26(23)	8(15)	34(20)	87(17)	2(33)	
**Class 3 (moderate SE)**	156(30)	105(30)	37(33)	14(25)	51(31)	156(31)	0(0)	
**Class 4 (low SE)**	139(27)	117(33)	17(15)	5(9)	22(13)	138(27)	1(17)	

In a multinomial logistic regression adjusted for age and number of weeks on ART, education, CD4 count and gestation at ART initiation were significantly associated with class membership (S4 Table). Women with lower CD4 counts at ART initiation were more likely to belong to Class 1 than other classes. Increased education predicted membership to Class 3, experiencing moderate levels of all SE, compared to Class 1, experiencing most or all types of SE. In addition, starting ART earlier in pregnancy was associated with higher probability of belonging to latent classes characterized by higher levels of SE (Classes 1 and 2).

### Side effects and ART adherence

At least one missed ART dose during pregnancy was reported by 32% of women (n = 166). Among these women, 87% (n = 145) reported between one and three missed doses, and the remaining 13% (n = 21) reported four or more missed doses, per 30 days on ART, respectively.(Table 2) Women who experienced any SE were significantly more likely to report any missed ART dose compared to those who reported no SE (100% versus 96%, p = 0.003).Women with any missed dose reported a marginally higher number of individual SE compared to women reporting no missed doses (median 5 versus 6 reported SE, p<0.001).

**Fig 3:**
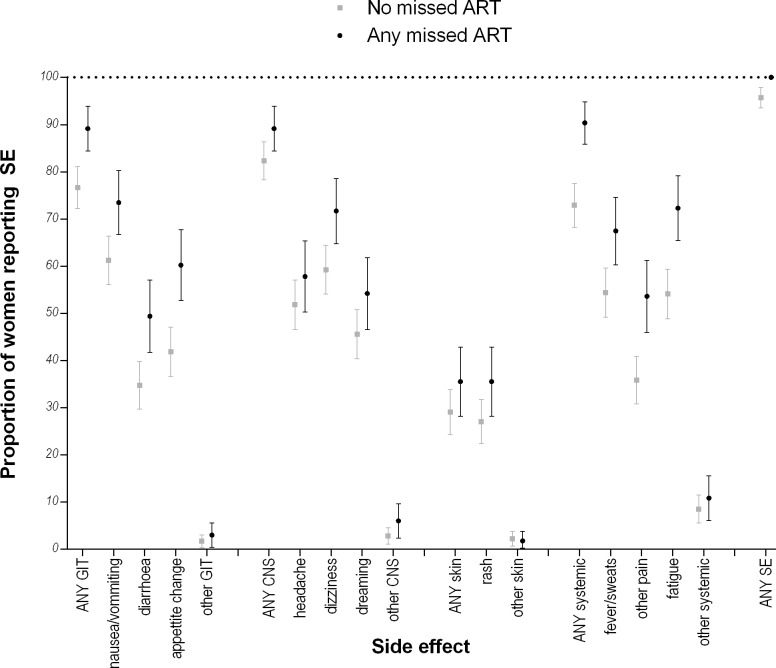
Proportions of women reporting each side effect with 95% confidence intervals, by any or no missed doses reported.

Fig 3 shows the proportions of women reporting individual SE grouped by reporting any versus no missed doses. For all systems SE groups, the proportion of women reporting SE was higher among those who missed any dose compared to those who reported no missed doses. These differences were statistically significant for all GIT, systemic and skin SE, except for the “other” categories which were reported at very low frequencies. Of the CNS SE, only dizziness was associated with reporting any missed ART doses. In the analysis of SE as latent classes, missed doses were reported more frequently in classes where SE reporting was more common: 36%, 21%, 31% and 13% of women who reported any missed ART during pregnancy were in Classes 1, 2, 3 and 4, respectively (p<0.001).(Table 2)

The associations between each SE construct (including frequency of SE, systems classification and latent classes) and missed ART doses were considered separately in multivariable logistic regression models adjusted for participant age, marital status, parity and time on ART. In all models, SE were consistently associated with missed ART doses (S4 Table). CD4 cell count, although associated with reporting SE, was not associated with missed doses and did not appear to confound the association between reported SE and missed ART doses. Higher numbers of different reported SE were associated with increasing odds of a missed dose (OR 1.20, CI 1.12–1.29, for each additional type of SE reported). Using systems SE categories, having any GIT or any systemic SE was associated with increased odds of reporting missed doses (OR 1.78 [CI 1.00–3.17] and OR 2.65 [CI 1.46–4.81], respectively). However CNS and skin SE were not associated with self-reported adherence. Compared to women in Class 1, women in Class 4 (with the lowest SE profile) had significantly lower odds of reporting any missed dose (OR 0.25, CI 0.14–0.45). These associations did not change appreciably when mode of delivery was included in the final model.

Six women (1%) reported missing 30 or more ART doses and the time off ART at assessment ranged from 43 to 156 days. The reasons reported for missing more than 30 days of ART are shown in S5 Table. Five out of six women were in Classes 1 or 2 where the experience of SE was highest, and four of the six women cited SE among the reasons for stopping to take their treatment.

## Discussion

Our study is the first to document very high levels of SE experienced by women initiating efavirenz-based ART during pregnancy in sub-Saharan Africa and an association with self-reported missed ART doses. Missed doses were reported frequently. However, we found no persistent associations between specific SE and missed doses; instead, the strongest associations with missed doses involved measures of the overall burden of SE experienced by women. This finding adds an important new perspective to ART adherence in pregnancy under Option B+.

Overall, 97% of women reported at least one SE and the vast majority of women reported multiple SE. While there are few data on ART SE reporting in pregnancy and methods of measurement vary, these levels appear substantially higher than in other studies of first line, EFV-containing regimens in non-pregnant adults.[7,17,46] As in other analyses of medication SE in pregnant women, it is not clear if the SE reported here were related to the antiretrovirals, routine supplements given in pregnancy (iron, folate and calcium), symptoms of pregnancy, or symptoms of HIV disease. Unfortunately we did not collect data on additional supplements however, use of over the counter supplements in this setting is not common. Concurrent tuberculosis medication may also impact reported side effects however less than 1% of this cohort had a tuberculosis episode during pregnancy. GIT and systemic SE may be related to physiologic changes of pregnancy, many of which typically occur early in pregnancy. The median gestation at ART start in this cohort was 21 weeks, meaning most women were well into the second trimester when they initiated ART. The finding that women with lower CD4 cell counts appeared to experience more diverse SE in both the absolute count and latent class analysis suggests that some of this reporting may be linked to HIV disease rather than ART use directly. There are concerns that healthier individuals starting ART may have more adherence problems. However, our findings show that individuals with lower CD4 cell counts are likely to experience more SE and therefore may also be at risk for poor adherence. These findings may have important implications for universal ART initiation in pregnancy as well as for adult “test and treat” approaches. Appropriate counselling and support for SE management is required both to prepare healthy individuals for the effects of ART, as well as to prepare the individuals with more advanced disease for the effects of their HIV and ART.

In general, EFV SE are thought to be relatively short-lived and improve over time. However, early SE on ART have not been well documented in the literature. Our findings suggest that women who booked for ANC later, and therefore had a longer duration on ART, report more SE than women with less time on ART. Further research on the timing and severity of ART SE during pregnancy and in non-pregnant women and healthy adults will be valuable to better understand these SE profiles and provide appropriate support in the early time on treatment.

Regardless of the underlying cause, all three constructs of reported SE (number of different SE reported, systems-based SE categories, and latent SE classes) were associated with reported missed ART doses. There was more imprecision around some associations between any SE or specific SE and missed doses observed in the logistic regression estimates in S4 Table, likely due to the very high proportions of women reporting SE in this cohort. In all three of the SE constructs used, effect sizes were relatively small and no specific SE appeared to be driving missed doses in any of the analyses. Although this may in part be due to inability to detect small differences with relatively homogenous reports of SE overall, this finding suggests that non-adherence may not be related to any one specific type or cluster of SE (for example nausea or dizziness), but rather to the overall SE burden.

This finding has several possible interpretations. The experience of multiple SE may present a cumulative burden of physical symptoms, any one of which may not contribute to non-adherence in isolation. There is evidence that the experience of SE is related to plasma antiretroviral levels, including EFV, which in turn may be moderated by genetic polymorphisms influencing drug metabolism.[47] The polymorphism resulting in increased plasma EFV levels has been found to be relatively common in patients of African descent, and high EFV concentrations have been associated with experiencing EFV related SE.[47, 48] In addition to this physiologic explanation, it is important to note that the reporting of larger numbers of SE may be more common in individuals with specific personality traits, such as neuroticism.[49] In this context it is plausible that mental health concerns may contribute to SE reporting as well as to non-adherence. These and other possible interpretations warrant additional attention.

This is the first report of SE experienced by pregnant women starting ART under Option B+ in South Africa. The data provide novel insights but are subject to important limitations. All women in this analysis were on a regimen of EFV+FTC/3TC+TDF, the regimen initiated by the majority of HIV-infected pregnant women under current global policy. However, we were unable to examine how this regimen compares to others with respect to reported SE and adherence. As individual antiretroviral agents have different SE profiles, the possibility that different ART regimens may have less SE and contribute to better adherence in pregnancy is a pertinent consideration. The prevalence of reported CNS SE, a specific concern in EFV-containing regimens, was very high and limited our ability to examine associations with non-adherence in this analysis.[11] More generally, all SE and adherence data was based on patient self-report, and social desirability may have led to misreports of ART adherence and/or reported SE, though we note the relatively high levels of missed dose reporting in this sample.

Despite these limitations, these findings have important implications for health services providing ART to pregnant women. Experiences of SE and fear of SE have been reported as barriers to adherence to ART in pregnancy.[3,20,22,23] The high level of reported SE and the association with missed doses found in this cohort supports previous calls for specific counselling on ART SE.[6,50] Antenatal ART initiation is often rapid to ensure maximum time on ART prior to delivery. Time for pre-ART counselling may be limited, however counselling to address possible ART SE, as well as pregnancy symptoms which may be perceived as ART related, should be a focus both at treatment initiation as well as subsequent follow up visits. More generally, the high burden of SE experienced in this cohort using EFV+FTC/3TC+TDF points to the ongoing need both to understand the causes of SE in context of pregnancy, and to identify new agents that minimize experience of SE.

In summary, these novel data are important in demonstrating a high level of SE experienced in this population of pregnant women initiating a first line EFV-containing regimen under Option B+. The overall burden of SE experience, rather than specific SE, appears persistently associated with self-reported missed ART doses. Although the reported SE may not be directly related to antiretroviral regimen, these data highlight that patient reported SE in the early time on ART may impact treatment adherence and there is a clear need to include counselling on expected ART SE at ART initiation and follow-up visits. In this analysis we did not find an association between CD4 cell count and missed ART doses which is reassuring for universal ART policies. As many countries move towards a “test and treat” approach, providing ART for all HIV-infected adults, the lessons we have learned from rapid universal ART initiation in pregnancy should be carefully considered. Managing experience and perception of ART SE may play an important role in optimizing adherence behavior during this early treatment period, which could in turn improve long term treatment outcomes.

## Supporting Information

S1 File Data set used for analyses. (XLSX)

S1 Table Comparison of the fit of Latent Class Analysis (LCA) models with different number of classes according to different criteria. (DOCX)

S2 Table Multinomial logistic regression model predicting latent class membership. (DOCX)

S3 Table Description of demographic and clinical characteristics according to the frequency of side effects (SE), systems-based SE categories, and latent SE classes. (DOCX)

S4 Table Logistic regression models predicting any missed dose. (DOCX)

S5 Table Reported reasons provided for missing 30 or more ART doses. (DOCX)
