# Supplementary material for: Self-Reported Side Effects and Adherence to Antiretroviral Therapy in HIV-Infected Pregnant Women under Option B+: A Prospective Study
Source: PLoS One. 2016 Oct 19;11(10):e0163079. doi: 10.1371/journal.pone.0163079 (PMC5070813; doi:10.1371/journal.pone.0163079)
Supplement: S1 Table — (DOCX) [file pone.0163079.s002.docx]

S1 Table. Comparison of the fit of Latent Class Analysis (LCA) models with different number of classes according to different criteria

| N | LL | AIC | aBIC | Entropy | BLR p | min SIZE % |
| --- | --- | --- | --- | --- | --- | --- |
| 1 | -3618.07 | 7258.13 | 7269.95 | 1 | - | 100% |
| 2 | -3323.78 | 6693.55 | 6718.25 | 0.75 | <0.001 | 47% |
| 3 | -3296.47 | 6662.94 | 6700.52 | 0.67 | 0.200 | 20% |
| 4 | -3278.07 | 6650.13 | 6700.60 | 0.74 | 0.016 | 17% |
| 5 | -3266.45 | 6650.89 | 6714.25 | 0.71 | 0.730 | 9% |

^N = Number of latent classes; LL = Log-Likelihood; AIC = Akaike's Information Criterion; aBIC=sample size adjusted Bayesian Information Criterion; Entropy = Classification Entropy (as calculated by Mplus, i.e. rescaled so that values closer to 1 indicate better discrimination); BLR p = p-value for the Bootstrap Likelihood Ratio test with null hypothesis that the fit of the model with n classes is not different from the fit of the model with n-1 classes (50 bootstrap draws).^

Models with one through five latent classes were compared in order to identify the number of distinct patterns of SE reported by women starting ART during pregnancy. The sample-size adjusted Bayesian Information Criterion (aBIC), which some simulation studies have shown to be superior to other indices in the LCA, reached its minimum with both the three- and four-class solutions, with no meaningful differences.[1] However, the lower value of the Akaike’s Information Criterion (AIC) and the results of the Bootstrap Likelihood Ratio test (BLR) both were in agreement, suggesting a better fit for the four-class model. Moreover, the four-class model was accompanied with higher classification entropy indicating a lower level of uncertainty in the classification of the individuals. The four-class solution was therefore selected to represent the data. The values of the various indices of fit used for the models and the results of the BLR test are reported here.

Reference:

1. Yang. Evaluating latent class analysis models in qualitative phenotype identification. Computational Statistics & Data Analysis. 2006; 50(4):1090-1104.
