# Supplementary material for: Self-Reported Side Effects and Adherence to Antiretroviral Therapy in HIV-Infected Pregnant Women under Option B+: A Prospective Study
Source: PLoS One. 2016 Oct 19;11(10):e0163079. doi: 10.1371/journal.pone.0163079 (PMC5070813; doi:10.1371/journal.pone.0163079)
Supplement: S2 Table — (DOCX) [file pone.0163079.s003.docx]

S2 Table. Multinomial logistic regression model predicting latent class membership

| **Class^§^** | **Predictor** | **A) Crude associations (n=517, except CD4, n=500)** | | | **B) Adjusted associations (n=500)** | | |
| --- | --- | --- | --- | --- | --- | --- | --- |
|  |  | **OR** | **(95% CI)** | **p-value** | **OR** | **(95% CI)** | **p-value** |
| **Class 2** | **One year increase in age** | 0.99 | (0.91-1.07) | 0.042 |  |  |  |
|  | **Socioeconomic status** |  |  |  |  |  |  |
|  | **Low** | (ref) |  |  | (ref) |  | - |
|  | **Middle** | 4.66 | (4.66-4.66) | <0.001 | 4.56 | (1.71-12.17) | 0.002 |
|  | **High** | 2.88 | (1.04-7.94) | 0.518 | 3.59 | (1.28-10.07) | 0.015 |
|  | **Married/cohabiting** | 0.67 | (0.32-1.4) | 0.379 | 1.19 | (0.51-2.79) | 0.680 |
|  | **Primagravid** | 2.68 | (1.07-6.71) | 0.469 | 1.71 | (0.65-4.49) | 0.277 |
|  | **1 unit increase in natural logarithm of pre-ART CD4** | 1.77 | (0.98-3.2) | 0.303 | 2.21 | (1.04-4.7) | 0.040 |
|  | **Diagnosed prior to pregnancy** | 0.52 | (0.25-1.1) | 0.380 | 0.66 | (0.28-1.58) | 0.351 |
|  | **ARV history** |  |  |  |  |  |  |
|  | **ARV naive** | (ref) |  | - |  |  |  |
|  | **Previous PMTCT** | 0.86 | (0.36-2.04) | 0.443 |  |  |  |
|  | **Previous ART** | 0.51 | (0.05-5.4) | 1.209 |  |  |  |
|  | **Increasing weeks gestation at ART start** | 1.11 | (1.06-1.16) | 0.024 | 1.12 | (1.03-1.22) | 0.010 |
|  | **Increasing weeks on ART** | 0.93 | (0.89-0.97) | 0.021 | 1 | (0.93-1.07) | 0.911 |
| **Class 3** | **One year increase in age** | 1.01 | (0.96-1.07) | 0.042 |  |  |  |
|  | **Socioeconomic status** |  |  |  |  |  |  |
|  | **Low** | (ref) |  | - | (ref) |  | - |
|  | **Middle** | 2.98 | (1.35-6.58) | <0.001 | 2.9 | (1.25-6.71) | 0.013 |
|  | **High** | 2.7 | (1.26-5.78) | 0.518 | 3.04 | (1.28-7.22) | 0.012 |
|  | **Married/cohabiting** | 0.53 | (0.28-0.99) | 0.379 | 0.76 | (0.38-1.52) | 0.437 |
|  | **Primagravid** | 1.18 | (0.5-2.81) | 0.469 | 0.71 | (0.27-1.9) | 0.499 |
|  | **1 unit increase in natural logarithm of pre-ART CD4** | 1.35 | (0.83-2.19) | 0.303 | 1.65 | (0.95-2.86) | 0.077 |
|  | **Diagnosed prior to pregnancy** | 0.66 | (0.36-1.21) | 0.380 | 0.68 | (0.34-1.36) | 0.274 |
|  | **ARV history** |  |  |  |  |  |  |
|  | **ARV naive** | (ref) |  | - |  |  |  |
|  | **Previous PMTCT** | 0.97 | (0.47-1.97) | 0.443 |  |  |  |
|  | **Previous ART** | 1.51 | (0.37-6.17) | 1.209 |  |  |  |
|  | **Increasing weeks gestation at ART start** | 1.03 | (0.98-1.08) | 0.024 | 1.02 | (0.96-1.08) | 0.575 |
|  | **Increasing weeks on ART** | 0.98 | (0.94-1.01) | 0.021 | 0.98 | (0.93-1.02) | 0.327 |
| **Class 4** | **One year increase in age** | 1.02 | (0.97-1.07) | 0.042 |  |  |  |
|  | **Socioeconomic status** |  |  |  |  |  |  |
|  | **Low** | (ref) |  | - | (ref) |  | - |
|  | **Middle** | 1.92 | (0.96-3.83) | <0.001 | 2.27 | (1.06-4.86) | 0.035 |
|  | **High** | 1.43 | (0.72-2.82) | 0.518 | 2.25 | (0.99-5.1) | 0.052 |
|  | **Married/cohabiting** | 0.7 | (0.4-1.24) | 0.379 | 0.93 | (0.48-1.81) | 0.829 |
|  | **Primagravid** | 1.12 | (0.49-2.53) | 0.469 | 0.99 | (0.42-2.32) | 0.974 |
|  | **1 unit increase in natural logarithm of pre-ART CD4** | 1.86 | (1.13-3.07) | 0.303 | 2.11 | (1.23-3.63) | 0.007 |
|  | **Diagnosed prior to pregnancy** | 0.67 | (0.38-1.18) | 0.380 | 0.79 | (0.41-1.52) | 0.489 |
|  | **ARV history** |  |  |  |  |  |  |
|  | **ARV naive** | (ref) |  | - |  |  |  |
|  | **Previous PMTCT** | 1.11 | (0.59-2.12) | 0.443 |  |  |  |
|  | **Previous ART** | 0.22 | (0.01-4.67) | 1.209 |  |  |  |
|  | **Increasing weeks gestation at ART start** | 1.11 | (1.06-1.16) | 0.024 | 1.07 | (0.99-1.16) | 0.097 |
|  | **Increasing weeks on ART** | 0.91 | (0.88-0.95) | 0.021 | 0.95 | (0.88-1.02) | 0.169 |

§ Compared to reference class 1
