# Supplementary material for: Self-Reported Side Effects and Adherence to Antiretroviral Therapy in HIV-Infected Pregnant Women under Option B+: A Prospective Study
Source: PLoS One. 2016 Oct 19;11(10):e0163079. doi: 10.1371/journal.pone.0163079 (PMC5070813; doi:10.1371/journal.pone.0163079)
Supplement: S3 Table — (DOCX) [file pone.0163079.s004.docx]

S3 Table. Description of demographic and clinical characteristics according to the frequency of side effects (SE), systems-based SE categories, and latent SE classes.

|  | **Median frequency of reported SE (IQR)** | **Any GIT SE** | **Any CNS SE** | **Any skin SE** | **Any systemic SE** | **Class 1 (high SE)** | **Class 2 (moderate SE, high systemic)** | **Class 3 (moderate SE, low systemic)** | **Class 4 (low SE)** |
| --- | --- | --- | --- | --- | --- | --- | --- | --- | --- |
| **All women** | 5(3-7) | 417 (81) | 437(85) | 161(31) | 406(79) | 133 (26) | 89(17) | 156(30) | 139(27) |
| **Median age (IQR)** | - | 27(24-31) | 28(25-32) | 28(24-31) | 27(24-31) | 28(24-31) | 27(23-31) | 28(25-32) | 28(25-33) |
| **Socioeconomic status** |  |  |  |  |  |  |  |  |  |
| **lowest** | 6(3-8) | 161(39) | 168(38) | 66(41) | 149(37) | 63(47) | 27(30) | 51(33) | 56(40) |
| **medium** | 5(3-7) | 120(29) | 127(29) | 43(27) | 121(30) | 32(24) | 34(38) | 47(30) | 39(28) |
| **highest** | 5(3-7) | 136(33) | 142(32) | 52(32) | 136(34) | 38(29) | 28(31) | 58(37) | 44(32) |
| **Education level** |  |  |  |  |  |  |  |  |  |
| **Finished high school** | 5(3-7) | 108(26) | 114(26) | 39(24) | 106(26) | 26(20) | 25(28) | 49(31) | 34(24) |
| **Did not finish high school** | 5(3-8) | 309(74) | 323(74) | 122(76) | 300(74) | 107(80) | 64(72) | 107(69) | 105(76) |
| **Employment status** |  |  |  |  |  |  |  |  |  |
| **Employed** | 5(3-7) | 151(36) | 159(36) | 58(36) | 152(37) | 46(35) | 38(43) | 55(35) | 54(39) |
| **Not employed** | 5(3-7) | 266(64) | 278(64) | 103(64) | 254(63) | 87(65) | 51(57) | 101(65) | 85(61) |
| **Relationship status** |  |  |  |  |  |  |  |  |  |
| **Married/cohabiting** | 6(3-8) | 160(38) | 178(41) | 62(39) | 152(37) | 60(45) | 34(38) | 52(33) | 52(37) |
| **Not married/cohabiting** | 5(3-7) | 257(62) | 259(59) | 99(61) | 254(63) | 73(55) | 55(62) | 104(67) | 87(63) |
| **Median gravidity (IQR)** | - | 2(2-3) | 2(2-3) | 2(2-3) | 2(2-3) | 2(2-3) | 2(1-3) | 2(2-3) | 2(2-3) |
| **Primigravida** | 6(3-7) | 82(20) | 75(17) | 23(14) | 77(19) | 21(16) | 25(28) | 25(16) | 22(16) |
| **Multigravida** | 5(3-7) | 335(80) | 362(83) | 138(86) | 329(81) | 112(84) | 64(72) | 131(84) | 117(84) |
| **Timing of HIV diagnosis** |  |  |  |  |  |  |  |  |  |
| **In the current pregnancy** | 6(3-7) | 230(55) | 236(54) | 90(56) | 179(44) | 65(49) | 54(61) | 88(56) | 79(57) |
| **Prior to this pregnancy** | 5(3-8) | 187(45) | 201(46) | 71(44) | 227(56) | 68(51) | 35(39) | 68(44) | 60(43) |
| **ARV history** |  |  |  |  |  |  |  |  |  |
| **ARV naïve** | 5(3-7) | 303(73) | 316(72) | 112(70) | 299(74) | 96(72) | 67(75) | 111(71) | 100(72) |
| **Previous PMTCT** | 5(3-7) | 100(24) | 104(24) | 40(25) | 92(23) | 32(24) | 20(22) | 37(24) | 37(27) |
| **Previous ART** | 6(4-8) | 14(3) | 17(4) | 9(6) | 15(4) | 5(4) | 2(2) | 8(5) | 2(1) |
| **Median CD4 cell count at ART initiation (IQR) ^§^** | - | 351(235-528) | 355(239-528) | 345(213-528) | 354(236-531) | 318(220-466) | 403(246-548) | 367(239-539) | 400(276-589)* |
| **CD4≤200** | 6(4-8)* | 78(19) | 75(18) | 33(22) | 75(19) | 29(23) | 13(15) | 28(19) | 15(11) |
| **CD4 201 - 350** | 6(3-8) | 122(30) | 130(31) | 45(29) | 116(29) | 43(34) | 24(28) | 42(28) | 41(30) |
| **CD4>350** | 5(3-7) | 202(50) | 217(51) | 75(49) | 203(52) | 56(44) | 50(57) | 80(53) | 79(59) |
| **Median gestation(weeks) at ART initiation (IQR)** | - | 21(16-26) | 20(16-26) | 21(16-26) | 20(16-26) | 19(15-23) | 24(19-30) | 20(15-26) | 23(18-29)** |
| **Median weeks on ART (IQR)** | - | 19(13-24) | 19(13-24) | 19(14-24) | 19(14-24) | 21(16-25) | 16(11-22) | 19(12-25) | 16(10-21)** |
| ^§^17missing CD4 counts |  |  | | |  | **denotes p<0.05, **denotes p<0.001 using Kruskal Wallis for continuous and chi-squared for categorical variables* | | | |
