# Supplementary material for: Self-Reported Side Effects and Adherence to Antiretroviral Therapy in HIV-Infected Pregnant Women under Option B+: A Prospective Study
Source: PLoS One. 2016 Oct 19;11(10):e0163079. doi: 10.1371/journal.pone.0163079 (PMC5070813; doi:10.1371/journal.pone.0163079)
Supplement: S4 Table — (DOCX) [file pone.0163079.s005.docx]

S4 Table. Logistic regression models predicting any missed dose

|  | **A) Crude associations (n=517, except CD4, n=500)** | | | **B) Adjusted associations using number of side effects (n=517)** | | | **C) Adjusted associations using system categories (n=517)** | | | **D) Adjusted associations using latent classes* (n=517)** | | |
| --- | --- | --- | --- | --- | --- | --- | --- | --- | --- | --- | --- | --- |
|  | **OR** | **(95% CI)** | **p-value** | **OR** | **(95% CI)** | **p-value** | **OR** | **(95% CI)** | **p-value** | **OR** | **(95% CI)** | **p-value** |
| **One year increase in age** | 0.95 | (0.92-0.99) | 0.005 | 0.97 | (0.93-1.01) | 0.105 | 0.97 | (0.93-1.01) | 0.125 | 0.97 | (0.93-1.01) | 0.094 |
| **Socioeconomic status** |  |  |  |  |  |  |  |  |  |  |  |  |
| **Low** | (ref) |  |  |  |  |  |  |  |  |  |  |  |
| **Middle** | 1.15 | (0.73-1.81) | 0.538 |  |  |  |  |  |  |  |  |  |
| **High** | 1.11 | (0.71-1.73) | 0.640 |  |  |  |  |  |  |  |  |  |
| **Married/cohabiting** | 0.72 | (0.49-1.06) | 0.097 | 0.72 | (0.47-1.09) | 0.116 | 0.77 | (0.51-1.16) | 0.213 | 0.73 | (0.48-1.11) | 0.137 |
| **Primagravid** | 1.60 | (1.01-2.54) | 0.047 | 1.21 | (0.71-2.09) | 0.482 | 1.17 | (0.68-2.01) | 0.575 | 1.16 | (0.67-2.01) | 0.593 |
| **1 unit increase in natural logarithm of pre-ART CD4** | 1.00 | (0.49-2.03) | 0.991 |  |  |  |  |  |  |  |  |  |
| **Diagnosed prior to pregnancy** | 0.96 | (0.66-1.39) | 0.825 |  |  |  |  |  |  |  |  |  |
| **ARV history** |  |  |  |  |  |  |  |  |  |  |  |  |
| **ARV naive** | (ref) |  |  |  |  |  |  |  |  |  |  |  |
| **Previous PMTCT** | 0.69 | (0.44-1.08) | 0.107 |  |  |  |  |  |  |  |  |  |
| **Previous ART** | 1.06 | (0.38-2.93) | 0.909 |  |  |  |  |  |  |  |  |  |
| **Increasing weeks gestation at ART start** | 0.98 | (0.95-1.01) | 0.113 |  |  |  |  |  |  |  |  |  |
| **Increasing weeks on ART** | 1.03 | (1.01-1.05) | 0.011 | 1.02 | (0.99-1.04) | 0.128 | 1.02 | (1.00-1.04) | 0.111 | 1.02 | (1.00-1.04) | 0.113 |
| **Any GIT SE** | 2.51 | (1.45-4.34) | 0.001 |  |  |  | 1.78 | (1.00-3.17) | 0.051 |  |  |  |
| **Any CNS SE** | 1.76 | (1.01-3.09) | 0.047 |  |  |  | 1.25 | (0.68-2.30) | 0.479 |  |  |  |
| **Any Skin SE** | 1.34 | (0.91-1.99) | 0.138 |  |  |  | 1.18 | (0.79-1.79) | 0.419 |  |  |  |
| **Any Systemic SE** | 3.48 | (1.97-6.13) | <0.001 |  |  |  | 2.65 | (1.46-4.81) | 0.001 |  |  |  |
| **Increasing no. of reported SE** | 1.21 | (1.12-1.30) | <0.001 | 1.20 | (1.12-1.29) | <0.001 |  |  |  |  |  |  |
| **Class 1 (high SE)** | 1 | (ref) |  |  |  |  |  |  |  | 1 | (ref) |  |
| **Class 2 (moderate SE, high systemic)** | 0.72 | (0.19-1.26) | 0.389 |  |  |  |  |  |  | 0.79 | (0.45-1.39) | 0.409 |
| **Class 3 (moderate SE, low systemic)** | 0.62 | (0.24-1.00) | 0.124 |  |  |  |  |  |  | 0.61 | (0.37-0.99) | 0.046 |
| **Class 4 (low SE)** | 0.17 | (0.04-0.31) | <0.001 |  |  |  |  |  |  | 0.25 | (0.14-0.45) | <0.001 |

**Panel D is adjusted for the probability of class membership*
