# Supplementary material for: Self-Reported Side Effects and Adherence to Antiretroviral Therapy in HIV-Infected Pregnant Women under Option B+: A Prospective Study
Source: PLoS One. 2016 Oct 19;11(10):e0163079. doi: 10.1371/journal.pone.0163079 (PMC5070813; doi:10.1371/journal.pone.0163079)
Supplement: S5 Table — (DOCX) [file pone.0163079.s006.docx]

S5 Table. Reported reasons provided for missing 30 or more ART doses.

|  | Reason for missing 30 or more ART doses |
| --- | --- |
|  | “I ran out of treatment and did not go back to get more from clinic, I did not have transport money” |
|  | “I feel **stressed**, that is why I'm not taking my treatment. I **vomit after taking the pill**” |
|  | “I ran out of treatment…I wanted to **avoid side effects**” |
|  | “Because of **side effects**. I **vomited and lost appetite**” |
|  | “I stopped taking treatment due to exams, because they **made me sleepy and weak**. After that I became lazy to collect, avoiding explaining why I defaulted” |
|  | “After being discharged at hospital [after delivery], I did not come back to fetch my treatment. I was very late for my scheduled appointment to collect treatment and was afraid thereafter to come again. There is no reason for not taking treatment and it is not hard to take.” |
